# Nerve Growth Factor and Related Substances: A Brief History and an Introduction to the International NGF Meeting Series

**DOI:** 10.3390/ijms18061143

**Published:** 2017-05-26

**Authors:** Ralph A. Bradshaw, William Mobley, Robert A. Rush

**Affiliations:** 1 Department of Physiology and Biophysics, University of California, Irvine, CA 92697, USA; rablab@uci.edu; 2 Department of Pharmacology, University of California, San Diego, La Jolla, CA 92093, USA; 3 Department of Neuroscience, University of California, San Diego, La Jolla, CA 92093, USA; williammobley7@gmail.com; 4 Department of Human Physiology, Flinders University, Adelaide, SA 5001, Australia

**Keywords:** growth factors, neurotrophins, cell signaling, receptors, central nervous system, development

## Abstract

Nerve growth factor (NGF) is a protein whose importance to research and its elucidation of fundamental mechanisms in cell and neurobiology far outstrips its basic physiological roles. It was the first of a broad class of cell regulators, largely acting through autocrine and paracrine interactions which will be described herein. It was of similar significance in establishing the identity and unique roles of neurotrophic factors in the development and maintenance of the peripheral and central nervous systems. Finally, it contributed to many advances in the elaboration of cell surface receptor mechanisms and intracellular cell signaling. As such, it can be considered to be a “molecular Rosetta Stone”. In this brief review, the highlights of these various studies are summarized, particularly as illustrated by their coverage in the 13 NGF international meetings that have been held since 1986.

## 1. Pre-Meeting History

**Figure 1:**
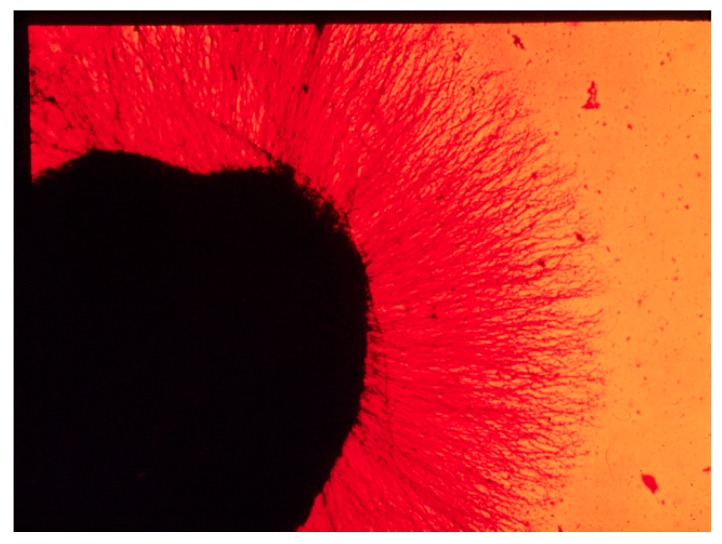
Nerve growth factor (NGF) “Halo” effect on cultured peripheral ganglia.

The discovery of nerve growth factor (NGF) is appropriately attributed to Rita Levi-Montalcini in the early 1950s, while she was working as a visiting scientist in the laboratory of Viktor Hamburger in St. Louis, Missouri. (Many fine reviews have been published over the past decades and represent a compendium of the history of NGF research, covering essentially all aspects of the chemistry and biology of it and its many related substances. References [1,2,3,4,5,6,7,8,9] are a sampling of such material and a good starting point for readers interested to read more details). However, it can be argued that the first experimental observations of its activity were made by Elmer Bueker with transplanted tumors (mouse sarcomas 37 and 180) a few years earlier [10]. Levi-Montalcini recognized that the hypertrophic effects exerted on the host (mouse) sympathetic neurons by the tumors was likely due to a soluble substance, in keeping with her previous proposals developed in Italy during the Second World War. Indeed, many years later when asked by one of us (Bill Mobley) when she knew she had discovered something uniquely different, Rita replied it was when she saw “axons invading blood vessels” for the first time. For her, this phenomenon marked a radical departure from the norm and pointed to a powerful new activity present. Its identification was the first step in revealing a new and very different story. To purify the activity, Rita devised an assay for the material, which she ultimately named NGF, (“Nerve growth factor” was actually not the first name given to it; rather it was originally termed nerve growth-promoting activity. However, this name was of short duration and was quickly supplanted by the growth factor designation. Stanley Cohen solidified this terminology when he named epidermal growth factor). This assay, developed in Rio de Janeiro in the laboratory of a colleague, Hertha Meyer, ultimately used explanted chick dorsal root ganglia in an inverted semi-solid plasma clot. Tumor-induced fiber growth radiated out to produce a halo-like effect and the original photographs are an iconic part of biological art (See Figure 1) [1]. While Levi-Montalcini went on to define the biological properties of this material, she worked in collaboration with Stanley Cohen to attempt the first isolation of NGF from the tumors [11]. Serendipitously, they identified snake venoms, then ultimately adult male mouse submandibular glands as considerably richer sources of NGF [12,13]. The latter provided sufficiently purified material to generate anti-sera. Cohen also showed that the submandibular tissue contained a second abundant substance, epidermal growth factor (EGF), which he eventually isolated and characterized [14]. EGF turned out to be at least as important as NGF to cell biology.The anti-mouse NGF proved effective in eliminating the development of the sympathetic nervous system (giving rise to the technique of immunosympathectomy), providing concrete proof of the biological significance of NGF [15] three decades before advances in molecular biology allowed for similar conclusions by using knockout animals [16].

With the identification of the submandibular gland source of NGF, two groups isolated the activity to apparent homogeneity, but surprisingly with quite different results. Eric Shooter’s group at Stanford and Silvio Varon’s group at University of California, San Diego, working in concert, described a high molecular weight complex, denoted 7S NGF, containing three types of polypeptides (termed α, β and γ) [17,18], while Piero Angeletti’s lab in Rome reported a smaller entity, which they called 2.5S NGF [19] (the S designations referring to their relative sedimentation coefficients). Only one of the subunits of the 7S complex had neurite-stimulating activity (βNGF), and its similar size to 2.5S NGF suggested that this was the principal biological entity, (the α and γ subunits were ultimately shown to be members of a large group of mouse glandular kallikreins that are essentially unique to that tissue and species. The γ subunit has proteolytic activity and causes the excision of a dipeptide from the C-terminus of the β subunit of mouse NGF but with no apparent physiological relevance). In 1971, Ruth Angeletti and Ralph Bradshaw determined that mouse 2.5S NGF was a non-covalently associated homodimer [20] and reported the monomeric amino acid sequence as a protein of 118 amino acids with three intrachain disulfide bonds [21]. In the following year, Bill Frazier analyzed both the primary structure and the known functional properties of NGF and concluded that it was significantly related to insulin. This suggested, for the first time, that NGF was an endocrine- or hormonal-like substance [22]. This hypothesis was to have far-reaching effects on endocrinology and cell biology. Prior to this speculation, endocrine substances such as insulin were thought to only travel systemically. Since it was already clear that NGF was not found in blood in sufficient amounts to meet this criteria, it was supposed that it was not endocrine in nature. For it to be related to a bona fide hormone meant that in all likelihood, the existing endocrine definitions were too limited. Indeed, at the same time as these studies on the chemical and biological properties of NGF were unfolding, several other substances were identified as additional growth factors (many bearing this designation). Several of these substances were ultimately key to expanding the concept that the endocrine system was much broader than originally defined, and included moieties with paracrine and autocrine actions. Importantly, in addition to other activities, many of these factors were either identified as neurotrophic agents or were shown to also have effects in the peripheral and/or the central nervous systems (PNS and CNS). The list of agents having neurotrophic activity now exceeds several hundred proteins and other biochemicals.

After the report of the insulin–NGF relationship appeared, three groups showed that responsive tissues bore NGF-specific binding entities, consistent with the presence of cell surface receptors (further cementing the case that they were endocrine-like in their action) [23,24,25,26]. The characterization of these entities, and their functional responses, over the next several years was significantly enhanced by the introduction of a cultured cell paradigm, the PC12 cell, by Lloyd Greene and Art Tischler [27]. A second observation that markedly enhanced the importance of NGF and its role in the nervous system was the demonstration that it was specifically taken up at the synapses of responsive peripheral neurons and retrogradely transported back to the soma of these cells by Ian Hendry, working in the laboratories of Iverson, Thoenen and Shooter. This observation provided a molecular mechanism to explain how neurons recognize that the correct synaptic junctions have been made [28,29].

Following the identification of NGF receptors on the cell surface of responsive tissues (mainly PNS neurons and PC12 cells but also in the CNS) using radiotracers and insolubilized ligand [23,24,25,26], various biochemical analyses by Bradshaw’s group established a molecular mass in the range of 130 kDa for the active entity [30,31,32]. However, these results were contradicted by reports of an NGF-specific receptor form of about half of that molecular weight [33,34]. The identification of the NGF receptor was further confounded by equilibrium binding studies that predicted both high and low affinity forms as well as negatively cooperative behavior [23,24,25,26,35]. In the mid 1980s, the lower molecular weight species was cloned by both Shooter’s and Moses Chao’s laboratories [36,37]. Its protein molecular weight was <50,000, but it behaved as a larger molecule on SDS-PAGE because of heavy glycosylation and was subsequently designated p75.

In 1981, the groups of Bill Rutter at University of California, San Francisco and Axel Ullrich at Genentech independently reported the cloning of the NGF cDNA. Their research showed that like other hormonal entities, NGF was synthesized as a larger precursor with a substantial prepro N-terminal extension [38,39]. This material also provided appropriate nucleic acid-based reagents to ascertain a more accurate picture of NGF tissue expression, and led to the identification of CNS activities, confirming earlier suggestions that its activity was not limited to the PNS [40].

Hamburger and Levi-Montalcini recognized that only a proportion of sensory neurons responded to the presence of NGF, but the existence of a second neurotrophic factor acting on the NGF-non responsive neurons was not demonstrated until just prior to the first NGF meeting in 1986. The work of several members of the Thoenen laboratory, most notably Yves-Alain Barde, led to the description of the biological activity of brain-derived neurotrophic factor (BDNF) and its homologous relationship to NGF [41]. Subsequently, cloning techniques expanded the family to four members with the addition of neurotrophins 3 and 4 [42,43].

## 2. The First International Meetings

**Figure 2:**
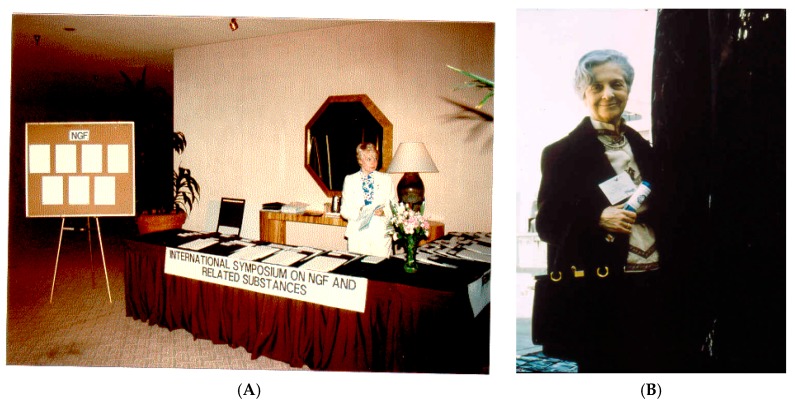
(**A**) Registration desk in Monterey for the 1st NGF conference held in 1986; and (**B**) Honoree, Rita Levi-Montalcini.

In the summer of 1985 in a lovely garden in Tokorozawa, Japan, Stanley Cohen and Ralph Bradshaw were taking a leisurely break from the meeting they were attending and were using the opportunity to reminisce about the early days of growth factor research. It was Stanley who noted that no one had properly fêted Rita Levi-Montalcini and a birthday party/symposium would be timely and appropriate. He calculated that she must be approaching her 75th birthday, which turned out to be off by a couple of years (she was already 76, so in the end it was a 77th birthday celebration). Nonetheless, the concept of an NGF meeting, the first of its kind, remained highly attractive. In due course, Bradshaw and Eric Shooter, organized the meeting with the extensive help of many others. At the suggestion of Eric Shooter, the conference was held in Monterey, California in April, 1986 (Figure 2). It coincided with Rita’s birthday and the final banquet was complete with a birthday cake and the expression of many best wishes. Parenthetically, Rita and Stanley received the Nobel Prize for their pioneering work the following fall.

The meeting covered a broad spectrum of issues that were at the forefront of growth factor research, from biosynthesis to function, with considerable discussion on related neurotrophic substances (albeit the complete neurotrophin family was yet to be discovered). Many presentations were focused on signaling as well as the recently discovered role for NGF in the CNS by Hefti in the Thoenen group, Mobley, and Reichardt; these topics have continued to be an important part of every meeting held thereafter. In summarizing the meeting, Rob Rush noted, “one suspects ‘Rita’s factor’ will continue to lead us into many more ‘unchartered routes’ over the next 40 years. Perhaps the organizers can be persuaded to keep us from dangerous waters by making this conference the first of many” [44].

**Figure 3:**
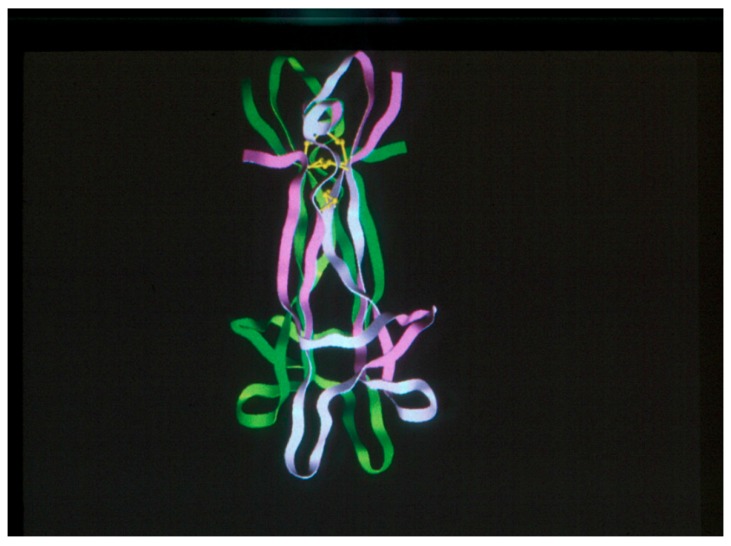
Crystal structure of NGF [45]. Shown at the 2nd NGF conference held in 1991.

In 1991, after a five-year hiatus, Rob Rush, with the help of Ian Hendry and Perry Bartlett, organized the second meeting in the series on Daydream Island in the Whitsunday Island chain of Australia as a satellite to the International Society of Neurochemistry conference held in Sydney the following week. Shortly before the meeting, the structure of βNGF had been determined by Neil McDonald, Risto Lapatto, Judith Murray-Rust, Jennifer Gunning, Alexander Wlodawer and Tom Blundell [45] and was shown to this group for the first time (in a slide provided by them and shown by Ralph Bradshaw) (Figure 3). The discovery of BDNF by Barde heralded the appearance of the neurotrophin family [41,42,43]. Of equal importance, it became clear that this meeting served a unique purpose for the greater neurotrophic factor research family and these meetings needed to continue. Researchers were quick to seek clinical applications for the neurotrophins, and Lars Olson reported on the first trial involving a single patient with Alzheimer’s disease at the next meeting [46]. Richard Murphy, then in Montreal, stepped up to organize this third meeting three years later at beautiful Lake Louise in Alberta, Canada and the series became firmly established. This edition was dedicated to Eric Shooter for his multiple contributions to the field and featured discussion of many important new aspects including the discovery and characterization of the Trk family of receptors, structure/function relationships in the NGF molecule (particularly with respect to ligand interactions with p75 and TrkA), and the processing of proneurotrophin precursors to their mature forms. However, the biological action of the proneurotrophins was not uncovered until more than a decade later.

## 3. The International NGF Meeting Series

**Figure 4:**
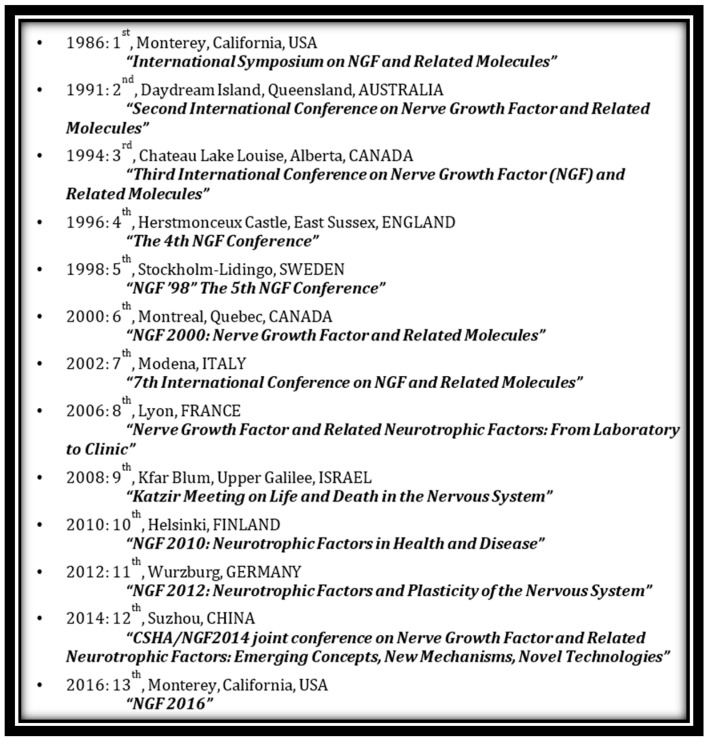
List of all NGF conferences held over 30 years 1986–2016.

Following the third meeting in 1994, the series became established on a biannual basis in a variety of global venues (see Figure 4). In 1996, it was held at Herstmonceux Castle outside of London, UK and honored Hans Thoenen. It also marked the steady shift of focus to more biological aspects of neurotrophic factors, including the introduction of some new players, while at the same time there was a decreasing emphasis on structure. The expansion of the field to new neurotrophic entities was actually the continuation of a trend that had started in the very first meeting when it was already clear that NGF was not a unique, singular moiety, but a member of a much larger group of substances that exerted various responses in both the peripheral and central nervous systems. Indeed almost every factor that has been identified has some role in the nervous system, in addition to other non-neuronal activities (for which it is often better known). The same can be said for substances like NGF, which is thought of as neuronal factor, but has many well-described non-nervous system functions.

The 5th meeting was held in Stockholm and featured the participation of Rita Levi-Montalcini for the first time since the original Monterey meeting. Although she did not attend the Millennium meeting in Montreal in 2000, she did make appearances at the 2002 meeting in Modena, Italy; the 2006 meeting in Lyon, France, (there was no meeting held in 2004); and the 2008 meeting in Kfar Blum, Israel [47]. The final four meetings were held in Helsinki, Finland; Wurzburg, Germany; Shanghai, China; and finally, in a return to the original venue, Monterey, California, (the original meeting was held in the Doubletree Hotel in the center of Monterey; the 13th was held at the Asilomar Conference Center a few miles away). Several meetings highlighted the research contributions of Stan Cohen, Alberta Aguayo and Gordon Guroff, and others programmed a special plenary lecture from other honorees including Bradshaw (at Suzhou, 2014) and Rush (at Monterey, 2016).

**Figure 5:**
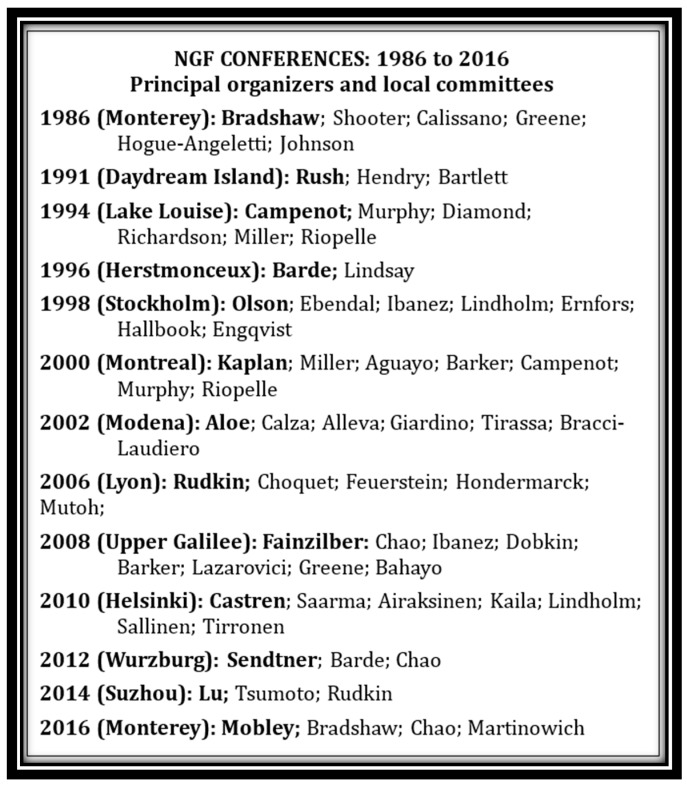
List of the complete 13 conferences in the NGF and Related Molecules series, 1986–2016, organizing committees and venue.

While universally recognized as important contributors to the neurotrophic factor field, the organizers have not only come from a broad geographic distribution, they also represent a crosssection of the scientific focus of scientists and physicians interested in these molecules (Figure 5). These range from chemists, biochemists and cell biologists to neuroscientists, neurologists, and even psychiatrists. It is indeed one of the unique features of the meeting series and of the field itself.

Although there has been an understandable shift of emphasis from structure and chemistry to function and biology and ultimately clinical aspects, as noted above, several themes have continued throughout that reflect their importance to the development of the field (see Table 1). These include signaling, the function of neurotrophic factors in the CNS, and identification and characterization of new factors. The programs of each meeting are too extensive to describe in detail, but they emphasize the continuous growth of the field.

**Figure 6:**
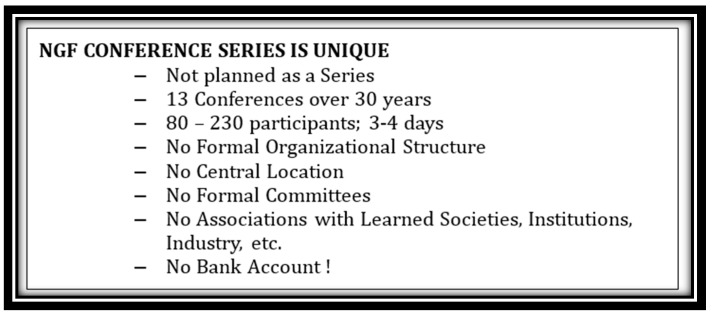
Characteristics that have made the NGF Conference series unique.

**Figure 7:**
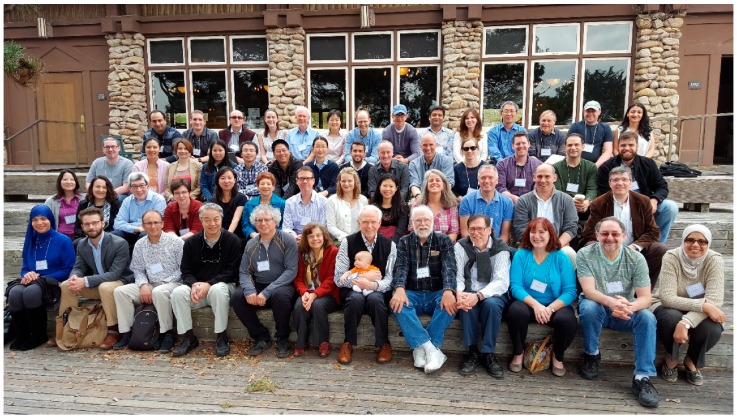
Attendees at the 13th NGF and Related Factors Conference, 30th ReUnion held in Monterey, April 2016.

The International NGF Meeting series has many unique features setting it apart from most scientific meetings (Figure 6). It did not start as a series and has little or no continuous leadership. With the exception of the 2004 meeting that ultimately was not held, there has been continued support, indeed enthusiasm, for the series. Considering it is not associated with any established organization or learned society, its overall stability and staying power is even more remarkable. The latest meeting (Figure 7), envisioned as a 30-year anniversary of the first meeting, continued the tradition and allowed some reflection on where the field has gone over the period between the two meetings [48]. The field has advanced enormously and continues to do so. Collectively and individually, the neurotrophic factor family, headed by the patriarch, NGF, has become central for revealing basic principles of cell and neurobiology and its relevance to disease—particularly those with their origins in the CNS, which are among the most devastating and intractable to manage and treat. There can be little doubt about the important role they have in these disorders and the potential they offer to provide routes to therapy.

In many ways, NGF has been a true Rosetta Stone in that it has provided insight into so many aspects of biology. As such, it rates as one of the most important discoveries in biology and it remains the gift that keeps on giving, much to the delight of those who have spent, or are spending or plan to do so, their careers pursuing and revealing its secrets.

NGF 2018 is scheduled to take place in Spain, June 23–26; once again a new venue that will certainly continue the traditions set by the previous 13 meetings.

## Figures and Tables

**Table 1:** Topics covered across 30 years of the NGF conferences. The yellow shading indicates topic was presented at the specific conference identified.

Location	Monterey	Daydream Island	Lake Louise	Herstmonceux	Stockholm	Montreal	Modena	Lyon	Upper Galilee	Helsinki	Wurzburg	Suzhou	Monterey
Topic/Year	1986	1991	1994	1996	1998	2000	2002	2006	2008	2010	2012	2014	2016
Biosynthesis/Chemistry													
Receptor Identification													
Signalling													
Development/Differentiation													
NTs in CNS													
Other Factors													
Connectivity													
Injury/Repair													
Therapeutics/Clinical Trials													
Regulation of Synthesis													
Non-Neuronal Functions													
Pain													
Stem Cells													
Memory/Behaviour													
Synaptic Function/Circuitry/Plasticity													
Pro Neurotrophins													
